# Quantum Dots Synthesis Through Direct Laser Patterning: A Review

**DOI:** 10.3389/fchem.2019.00252

**Published:** 2019-04-17

**Authors:** Francesco Antolini, Leonardo Orazi

**Affiliations:** ^1^Photonics Micro and Nanostructures Laboratory, Physical Technologies for Safety and Health Division, Fusion and Technologies for Nuclear Safety and Security Department, ENEA, Frascati, Italy; ^2^Department of Sciences and Methods for Engineering, University of Modena and Reggio Emilia, Reggio Emilia, Italy

**Keywords:** semiconductor quantum dots (QDs), precursors, direct laser patterning (DLP), QD-LEDs, display

## Abstract

In this brief review the advances on Direct Laser Patterning (DLP) for the synthesis of photo-luminescent semiconductor quantum dots (QDs) belonging to II-VI groups, especially in solid state using laser-assisted conversion are reported and commented. The chemistry of the precursor synthesis is illustrated because it is a crucial step for the development of the direct laser patterning of QDs. In particular, the analysis of cadmium (bis)thiolate and cadmium xanthates precursors after thermal and laser treatment is examined, with a special focus on the optical properties of the formed QDs. The second part of the review examines how the laser parameters such as the wavelength and pulse duration may regulate the properties of the patterned QDs. The DLP technique does not require complex laser systems or the use of dangerous chemical post treatments, so it can be introduced as a potential method for the patterning of pixels in quantum dot light emitting diodes (QD-LEDs) for display manufacturing.

## Semiconductor Quantum Dots (QDs) Synthesis and Properties

Colloidal photoluminescent semiconductor nano-crystals, also referred to as quantum dots (QDs) (Grim et al., 2015; Panfil et al., 2018), have received considerable interest both from the point of view of fundamental materials research and in terms of industrial needs especially in photonics application. The growing interest in this field is due to the unique optical and electronic properties of QDs including narrow emission, broad absorption, and high photo-stability compared with the corresponding bulk materials. Accordingly, QDs have great potential for several industrial applications, such as optoelectronic devices, LEDs (Yang et al., 2015), photovoltaics (Yang et al., 2017), lasers (Supran et al., 2013), sensors (Cho et al., 2015), and bio-labeling (Woo et al., 2013).

The optical properties of this material are determined by the mobility of hole and electrons confined in nanosized structures (Rossetti et al., 1984; Brus, 1986), and this phenomenon is called quantum size effect. Due to this effect it is possible to modulate the QDs optical properties by changing their size and/or shape, and it is possible in principle to get any color of the visible spectrum by tuning the particles to the right size. This means that by controlling the condition of the synthesis it is possible to control the absorption and emission spectra of the QDs.

Various chemical synthetic routes for semiconducting quantum dots have been previously reported: in organic solvents (Yu and Peng, 2002), in aqueous media (Lesnyak et al., 2013), the “one pot mixture” more suitable for the preparation of large amounts of QDs (Williamson et al., 2015) or even in polymers (Xu et al., 2015).

In the synthesis of QDs, a very important role is played by the passivation of the QDs core. Indeed the efficient confinement of the exciton (separation of hole and electron) within the core of the QDs and the absence of non-radiative phenomena is achieved only if the surface of the QDs is well-passivated (Klimov, 2010). Effective passivation can be achieved by controlling the surface chemistry of the QDs by over-coating the QD's surface with organic or inorganic shells it is possible to obtain luminescent quantum yield higher than 80% with narrow emission spectra (Hines and Kamat, 2014). The QDs stability, both over the time and under external conditions (light, oxygen, and moisture), can also be improved by modifying their surface properties through the introduction of organic ligands or inorganic “shells” to the structures.

With the advances in synthetic procedures, coupled with the development of surface modification techniques, the optical properties of QDs has improved to the point where they are now attracting significant interest for light-emitting applications (Shirasaki et al., 2013; Supran et al., 2013; Talapin and Steckel, 2013; Kathirgamanathan et al., 2015). The main optical properties showed by the QDs can be summarized in the following concepts: color purity, color tunability, bright emission, and stability.

Color purity and tunability are a consequence of the size quantization effect, which means that the nanocrystals show different absorption and emission spectra as a function of size and shape. The possibility to control their band-gap in a wide range gives rise to the tunability of color emission. The perception of color purity of the nanocrystals is related to the width of the emission spectra, which in turn is a function of the QDs size distribution. As the variance of QD's size distribution decreases, the full width at half-maximum (FWHM) of the emission band narrows. The typical values of the FWHM of a QD solution are 20–35 nm, resulting in luminescent sources with saturated emission colors (high color purity).

Another interesting feature of QDs is that their absorption gradually increases at shorter wavelengths. Such absorption spectra allow for simultaneous excitation of QDs with different emission color using a single blue pumping light. For these reasons QDs have found application in several fields including LEDs (Talapin and Steckel, 2013), solar cells (Jun et al., 2013), field-effect transistors (Choi et al., 2016), and for *in vivo* and *in vitro* imaging sensing and labeling techniques (Valizadeh et al., 2012), where their narrow emission linewidth, efficient luminescence, and broad absorption spectra give them an advantage over organic dyes.

## Patterning Strategies of QDs

The low-energy solution-based synthesis of QDs enables their scalability and incorporation into devices (processability) (Kathirgamanathan et al., 2015). Owing to colloidal stability and the ability to make films without disrupting the physical integrity of the crystals, the use of QDs in electroluminescent devices has become possible. Deposition techniques such as spin-coating, micro-contact printing, ink-jet printing, can be exploited to manufacture optoelectronic devices such as light emitting diodes (LEDs) and displays onto rigid or flexible substrates.

The manufacturing of displays in particular needs to pattern red-green-blue emitting QDs side-by-side at high spatial resolution. Commercial displays are formed by a matrix of a Red, Green, and Blue (RGB) areas, forming a pixel, that consecutively form the screens we are using in many devices. Therefore the industrial development of a QDs based display needs the development of an efficient way to pattern the QDs into an RGB matrix (Wang et al., 2017).

The laboratory fabrication of patterned quantum dot light emitting diodes (QD-LEDs) is mainly achieved by using spin-coating, which has some drawbacks for industrial applications. Indeed the material loss during the process is large at more than 90% (Haverinen et al., 2010) and the process cannot be used to make a multicolour pattern on a single substrate, which is a key step to developing a display.

For QD-LED displays to be commercialized, it is therefore necessary to develop manufacturing techniques to pattern different QDs based materials with (i) high spatial resolution and overall accuracy, (ii) high homogeneity, and (iii) high production rate for large scale production.

**Figure 1:**
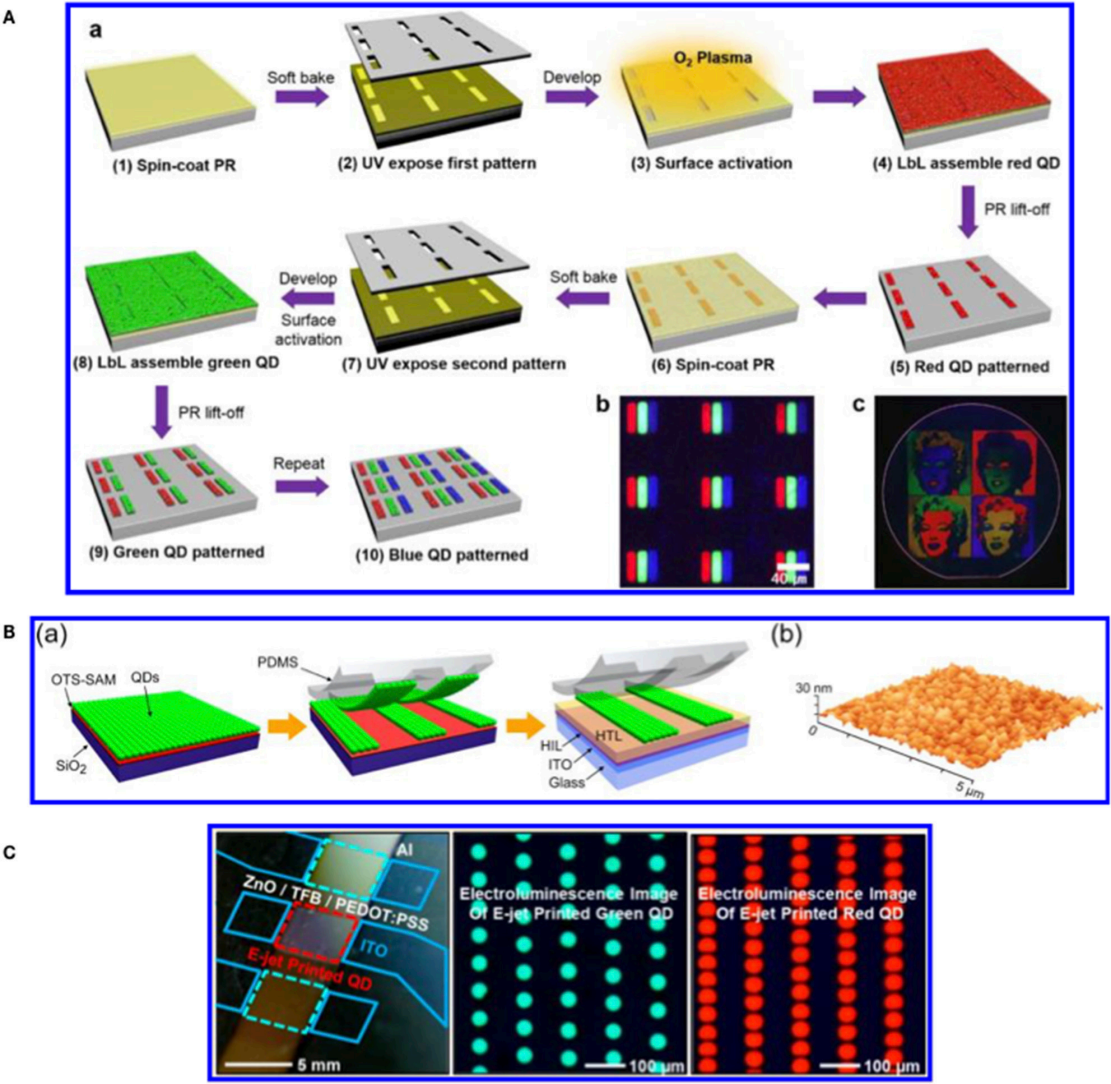
**(A)** Scheme of the photolithography steps for the realization of QDs display reprinted with permission from Park et al. (2016) copyright 2016 American Chemical Society. **(B)** Diagram of the CT steps for the realization of patterning on a QD-LED reprinted with permission from Cho et al. (2015) copyright 2015 American Chemical Society. **(C)** Ink Jet Printed green and red QDs for QD-LED realization reprinted with permission from Kim et al. (2015) copyright 2015 American Chemical Society.

Currently several techniques can be used to pattern QDs over different substrates and can be grouped in three main classes (Figure 1):

1. Photolithography (PLG)
2. Contact Transfer (CT)
3. Inkjet Printing (IJP).

Photolithography (Mack, 2008) is the dominant manufacturing approach for inorganic electronics and optoelectronics. The photolithography technique for QDs display manufacturing has been shown by Ji et al. (2018) and Park et al. (2016) (Figure 1A). In both works the photolithographic process is the same but the difference is that Ji deposited the QDs by spin coating while Park et al. used the layer by layer deposition at micron resolution. Table 1 summarizes the main characteristics of the photolithography.

**Table 1:** Resolution and characteristics of the most common patterning techniques.

**Patterning technique**	**Resolution**	**Pro**	**Cons**
Photolithography	<100 nm	Well-established, good resolution	Many preparation steps
Molding	<100 nm	Deposition on different substrates, good resolution	Many preparation steps
Ink jet	10–20 μm	Single step, large area	Low resolution

A similar approach based on the use of mask elements for QD-LED manufacturing is the mist deposition (Pickering et al., 2012). In this technique the QDs of different colors are deposited by dispersing the liquid in small droplets that pass through different registered shallow masks obtaining a matrix of alternating pixels (Zhu et al., 2008). This method allows the deposition of QDs solutions without damaging the previously deposited layers obtaining close packed pixels in the micron range.

Displays can be realized also by using Contact Transfer (CT) patterning (Cho et al., 2015; Dai et al., 2017). This technique is also called as molding or stamp printing. Here the basic idea is to use soft elastomeric stamps to replicate patterns generated by photolithography or other patterning techniques (Figure 1B). The process is obtained in two stages: in the first one the solution containing the QDs is deposited onto a substrate (the donor) obtaining a film. The film is then transferred from the donor substrate to a poly-dimethylsiloxane (PDMS) structured stamp (receiver) by pressing. The QDs are finally transferred from the PDMS stamp to the device. The mold can be used to create structures or to print organic materials over different substrates without using different solvents or particular functionalisation of materials (Dai et al., 2017). The molding methods have a good resolution (below 100 nm) however they need several steps in stamp preparation and chemicals to obtain the final device (Table 1).

The patterning by printing means that the active materials are deposited by physical contact through the use of nozzles (e.g., inkjet printheads, Wood et al., 2009; Haverinen et al., 2010; Teichler et al., 2013). The main characteristics of the printing methodology are (i) very low materials waste and (ii) easy modification of the desired pattern via software. Both characteristics contribute to lower the production costs especially for small batches. On the other side the challenges faced by ink jet printing rely on the development of suitable inks that avoid the nozzle clogging, the solvent evaporation (coffee ring effect) (Jiang et al., 2016) and improve the adhesion to the substrate (Cobas et al., 2015). The IJP is a simpler technology with respect to contact transfer and photolithography but on the other side its resolution is poorer (10–20 μm) (Menard et al., 2007) (Table 1). A recent development in IJP has been the introduction of electro-dynamic ink jet printing (Park et al., 2010). This improved method does not require any chemical or physical pre-patterning of the substrate, and can achieve a level of lateral and vertical resolution at the submicron level. By using this variant of the IJP, the Rogers's team (Kim et al., 2015) reported the deposition of red and green QDs dispersed in organic solvents and the manufacturing of a QD-LED.

The IJP method is technologically appealing indeed JOLED (Japan OLED) in 2017 announced the production of 21.1″ 4K OLED monitors panels with Ink Jet Printing technology (https://www.oled-info.com/joled, accessed October 2018).

## Direct Laser Patterning of QDs

A potential alternative to lithographic and printing techniques is the direct fabrication or direct patterning of QDs by means of a laser. The direct laser patterning (or laser writing) refers to the ability to produce arbitrary and programmable patterns of materials in a single step (or as a few as possible) by using a laser, with a medium resolution (<5 μm) and with an easy implementation in an industrial environment (Hocheng et al., 2014).

Lasers are unique tools that can be used to modify the morphological, physical, or chemical properties of surfaces without any mechanical contact. Lasers are widely used in the industry because they can clean or sinter the surfaces, ablate (drill, cut) and print. Laser printing has been introduced to transfer the active materials from a sacrificial layer to a substrate and can be considered as a particular printing method called Laser Induced Forward Transfer (LIFT) (Wolk et al., 2004; Feinaeugle et al., 2012).

Here the Direct Laser Patterning (DLP) is referred to the use of a laser as a tool to induce a chemical reaction, thus forming the desired product onto the substrate. This use is the one that is reported in the following sections and in particular for the synthesis of QDs and their surface modification.

### Materials for Direct Laser Patterning of QDs

The exploitation of laser direct patterning technology for the synthesis of QDs in solid state needs the development of suitable materials. Indeed, the molecules have to pick up the energy coming from the laser source and reorganize their structure to form QDs.

The type of molecules that can be used for laser direct patterning are the single source precursors (SSPs) that were successfully employed in the fabrication of crystalline and stable nanoparticles (Malik et al., 2010). These molecules are good candidates for direct laser patterning because the preformed bonds between the metal atom and the chalcogenide lead to the formation of nano-crystals with fewer defects. In addition, considering that the process takes place in solid state and the mobility of the atoms is reduced, in SSP the atoms are already “close” to their final position and the QDs growth can be facilitated.

In recent years raised the idea that molecules which in solid states give the nanocomposites by effect of their thermal decomposition could give QDs by the effect of laser (Antolini et al., 2006). This area of research was explored to study the formation of the II-VI QDs by several authors (Fragouli et al., 2010a; Resta et al., 2012; Agareva et al., 2015; Bansal et al., 2015).

The general approach for the evaluation of a precursor as possible candidate for laser patterning is its capacity to form QDs by thermal decomposition in solution or, better, in solid state. Some of the SSPs used for laser patterning were first evaluated for the generation of the QDs in solid state within a matrix by thermal decomposition (Antolini et al., 2005; Leventis et al., 2010; Resta et al., 2010; Dowland et al., 2011; Bansal et al., 2016), which is a common process to obtain nanocomposites.

**Figure 2:**
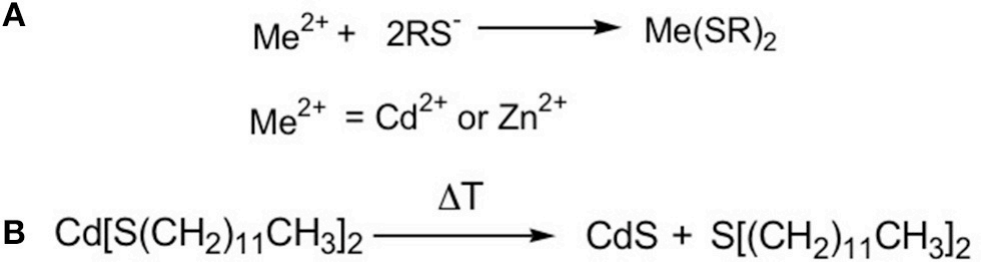
Scheme of the reactions for CdS synthesis: **(A)** The thiol solubilized in ethanol is added to the aqueous solution of the metal that form the metal bis thiolate; **(B)** By thermal treatment the cadmium thiolate is decomposed forming CdS QDs and a thioether.

The first molecule studied as precursor of CdS QDs under laser treatment was a chalcogenolate complex of cadmium, namely the cadmium (*bis*)dodecanthiol (Figure 2). This molecule was used to obtain CdS nanocomposite when dispersed in polystyrene (Antolini et al., 2005) and was selected due to its relatively easy chemical synthesis and stability in normal ambient conditions. The synthesis of this precursor, described in the literature (Rees and Kräuter, 1996), consists of a single step process: the metal salt is mixed in water/ethanol solution then the dodecanthiol, dissolved in ethanol, is added and the desired metal thiolate is formed as white precipitate (Figure 2A). This compound, once added to the polymer and heated at the proper time and temperature (300°C 10–15 min), produces the CdS QDs (Antolini et al., 2007, 2012) according with the reaction reported in Figure 2B.

When the loaded polystyrene film is treated with a laser at the wavelength of 1,064 nm, it is possible to observe the formation of a yellow area, like in the case of thermal treatment, which is characterized by the presence of the CdS QDs (Antolini et al., 2006). Here the effect of the laser beam is an increase of the temperature within the polystyrene film, caused by the absorption of a small fraction of the energy of the 1,064 nm laser source (see next paragraph). The formation of the QDs was revealed by the typical PL emission and absorption spectra. The PL linewidth, however, is quite large suggesting the presence of many surface defects as in the case of thermal treatment of the composite (Antolini et al., 2007).

The Athanassiou research group treated the same compound with two different laser sources at 266 nm and 355 nm, because these wavelengths match the absorption spectrum of the compound, so that the laser energy can be adsorbed directly by the precursor (Fragouli et al., 2010b). In these works Fragouli et al. (2010b) showed the importance of the choice of laser wavelength for the QDs formation in a PMMA matrix. Indeed, by using a laser source at 266 nm the resulting PL spectrum shows a broad emission centered at about 560 nm, while in the case of a similar treatment carried out with a laser source at 355 nm, it displays a sharper emission at about 500 nm. The emission broadening suggests the presence of many surface defects when the 266 nm laser source is used. The formation of the defects over the CdS QDs is caused by the higher reactivity of the PMMA under the 266 nm radiation. Indeed, the PMMA is damaged by the 266 nm wavelength and its decomposition products react with the growing QDs stimulating the formation of the QDs surface defects. When the used laser is at 355 nm, where the PMMA is transparent, the decomposition compounds are not created and the PL emission of the CdS QDs is sharper (Fragouli et al., 2010b). The effect of the presence of the PMMA, under 266 nm laser (15 ns pulse) treatment, was studied by the Bityurin group. Agareva et al. (2015) observed that without the PMMA the PL emission of the generated CdS QDs was always centered at about 600 nm independently from the number of pulses (from 1 to 50) and laser fluence (from 150 to 290 mJ/cm^2^). On the contrary when the laser treatment was carried out on PMMA loaded with the precursor the PL maxima of the formed QDs are blue shifted (from 600 nm to about 500 nm) from low to higher laser fluences (from 150 to 290 mJ/cm^2^). This suggests that the presence of a matrix influences the amount of the defects during the QDs growth.

The effect of both wavelength is not only the formation of the QDs but also their size modulation. Indeed Fragouli et al. (2009) observed that in both conditions the PL emission is red shifted by increasing the number of pulses. This observation suggests that enhancing the deposited energy the QDs size increases. The main difference between the two laser wavelengths is that at 266 nm the QDs are formed faster than at 355 nm. This effect is due to the different level of absorption of the radiation from the precursors that at 266 nm is high while at 355 nm is low.

**Figure 3:**
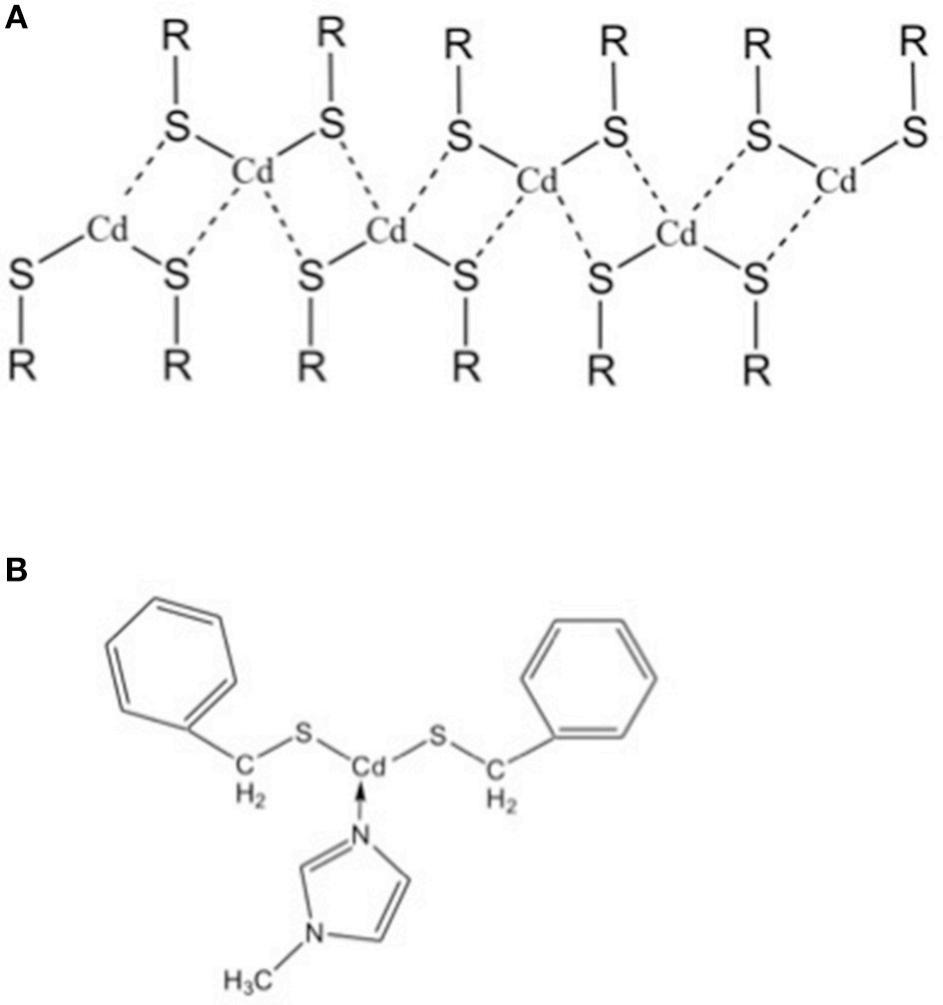
**(A)** Inorganic polymer structure of cadmium bis-thiolate. **(B)** Molecular formula of cadmium bis benzilthiolate functionalised with the 1-N-methylimidazole that avoid the formation of the inorganic polymer showed in “a,” because it blocks the free orbitals for the coordination with sulfur atoms of the neighboring molecules.

However, the main drawback of this Cd-based system lies in the chemistry of the precursor, because it is insoluble. Its insolubility is a critical point for the practical application of the laser patterning of QDs, because the QDs formation and their optical properties would be not homogeneous through the film itself and then the spatial resolution of the patterning would be decreased. The insolubility of the cadmium *bis*(dodecathiolate) is due to the formation of inorganic polymeric structures of the precursor, as a consequence of the strong bonds between the Cd atom and the lone pairs of sulfur atoms of the neighbor molecule (Figure 3A) (Dance, 1986). In general, these inorganic chains can be broken blocking the cadmium free orbitals using suitable ligands. In the particular case of the cadmium (*bis*)thiolates this issue was solved from the group of Tapfer (Resta et al., 2010) by introducing a new cadmium bis thiolate molecule, namely cadmium *bis*(benzilthiolate), functionalised with the 1-N-methylimidazole (MI). The role of the MI ligand is to block the free Cd orbitals with the nitrogen lone pairs beard by the MI so that the molecule cannot coordinate with its neighbors (Figure 3B). The molecule inside the polymer will interact only by weak chemical interactions such as Van der Waals forces, that can be broken by the organic solvents.

This precursor was successfully used by the Tapfer group to study more deeply the correlation between the energy deposited by the laser on the polymer/precursor film and the nanoparticle defects within the PMMA matrix (Resta et al., 2012). In this work the sample is treated with a 355 nm laser source changing in a systematic way the laser fluence and number of pulses. The results showed that the laser fluence drives the number of QDs while the number of pulses regulates the broadening in the size distribution. The laser fluence can be regarded as the temperature of annealing that determines the nucleation of the QDs while the total number of pulses modifies the size and shape distribution. Indeed, at higher fluencies the PL signal at about 395 nm is more intense, suggesting the presence of a higher number of QDs. When the number of pulses are increased under the same lasing conditions, the low energy emission band at about 500 nm increases, suggesting the defect formation.

A clear correlation between the laser energy deposited in the film and the particle size modulation was reported in the work of Camposeo et al. (2012). In this work a femtosecond laser source operating at around 800 nm was used and the energy delivery to the sample was mediated by a multi-photon process. Here it is shown that the PL emission is red shifted by increasing the fluence of the laser treatment. The PL emission of the QDs is formed by two contributions: the band edge emission centered at about 450 nm and the defects emission between 550 and 600 nm (shallow defects and deep trap states). Starting from the red shift of the band edge emission it is possible to calculate the size of the QDs as a function of the fluence: it was found that the QDs size changes from 5 to 8.5 nm varying the fluence from 0.15 to 0.45 J/cm^2^.

The solubility of the precursors is not the only chemical parameter to take into account in view of their application for a device manufacturing; indeed the decomposition temperature is another key parameter. The cadmium *bis*-dodecanthiol has a decomposition temperature of about 300°C that is too high when the decomposition is carried out within a polymer, because the organic matrix can decompose or be damaged. In addition, if the thermal/laser treatment has to be carried out in an electrical-driven multi-layer device, severe treatment conditions can damage the overall system. So it is necessary to explore other classes of molecules that decompose at lower temperatures as possible candidates for laser patterning.

**Figure 4:**
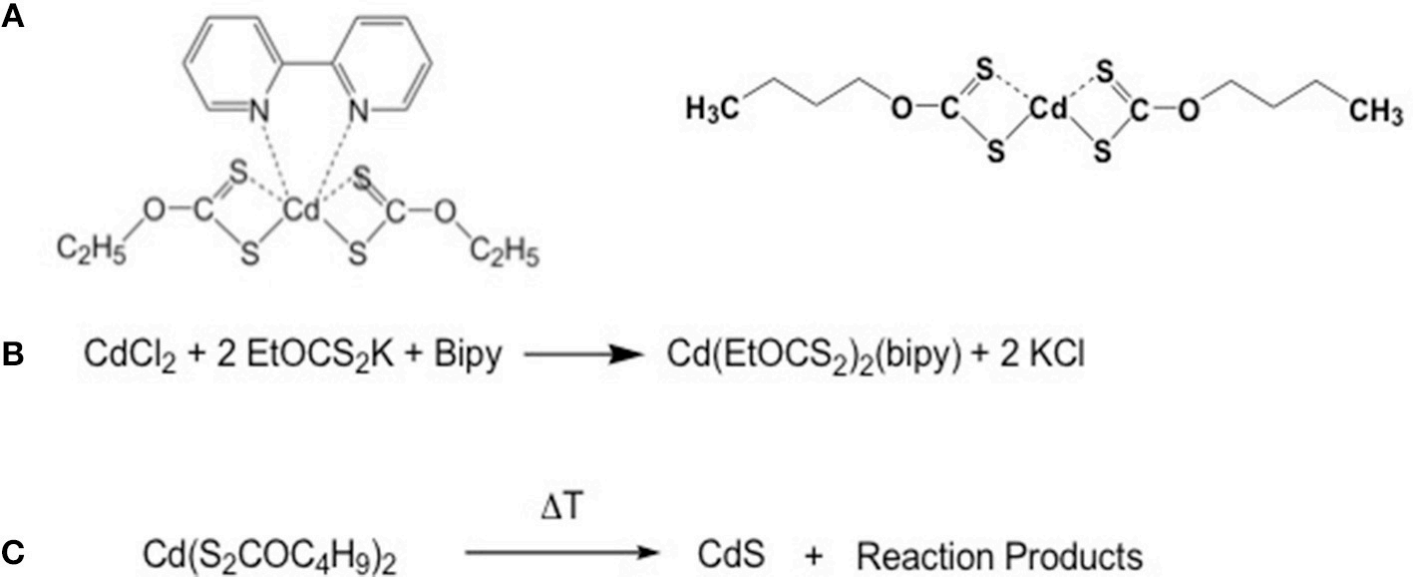
**(A)** Structural formula of cadmium diethylxantahte and dibuthylxanthate; **(B)** Scheme of the synthesis of cadmium diethyl xanthate; **(C)** Decomposition scheme of the cadmium diethylxanthate.

The xanthate molecules are another class of molecules that could be exploited for laser patterning because they can be easily solubilised in organic solvents and have decomposition temperatures below 200°C (Figure 4A) (Stroea et al., 2015). The use of xanthate molecules for QDs production has been proposed by Pradhan et al. (2003). The xanthates are characterized by the bond between the oxygen and the carbon which in turn is linked to the sulfur that coordinates the Cd atom. In the case of cadmium ethylxanthate, the bipyridil molecule is added to improve its solubility. The use of longer alkyl chains, like the buthyl chains, improves the solubility of the xanthates, that are preferred to form CdS QDs (Figure 4B) (Stroea et al., 2015).

**Figure 5:**
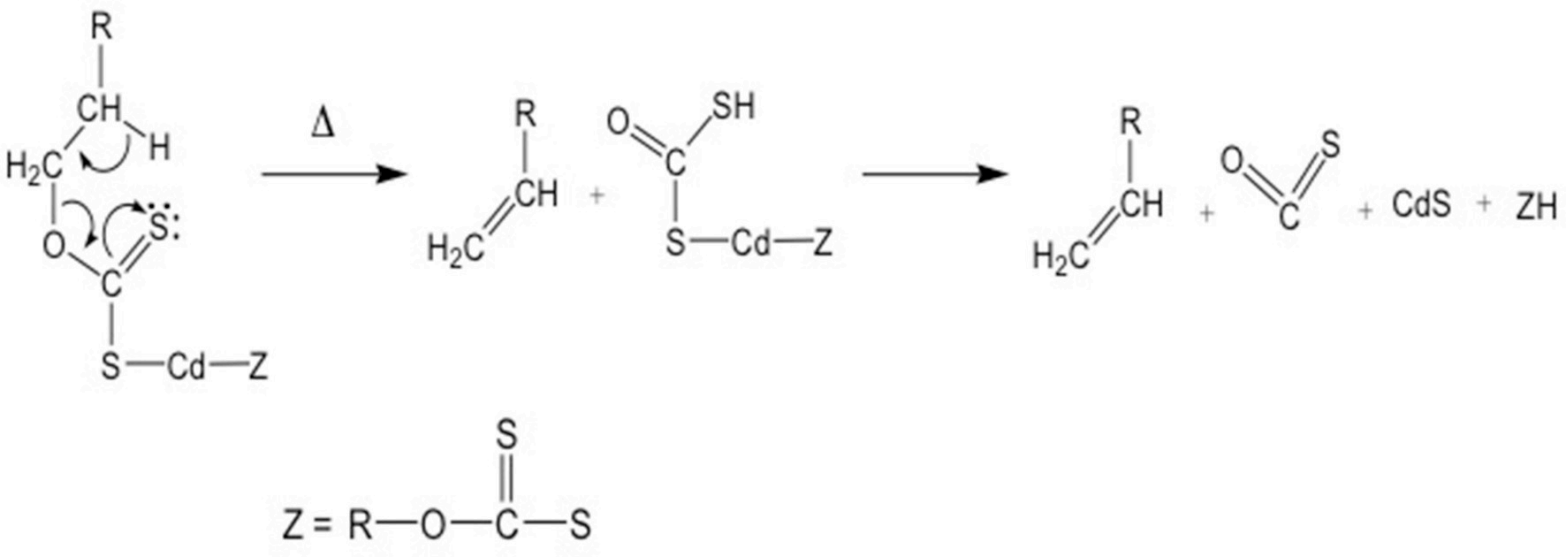
Decomposition path of the xanthate metallorganic compounds following the Chugaev mechanism.

These molecules present another interesting advantage: indeed their thermal decomposition gives rise to CdS QDs, while the reaction products undergo to fragmentation with the final formation of volatile compounds (Figure 5) (Pradhan et al., 2003).

This behavior is important, because in the case of QDs formation within a matrix or a device, no other compounds apart QDs should be present in the medium. In Figure 5 the OCS molecule and the olefin are the volatiles compounds leaving the matrix (Chugaev mechanism) during the heating.

**Figure 6:**
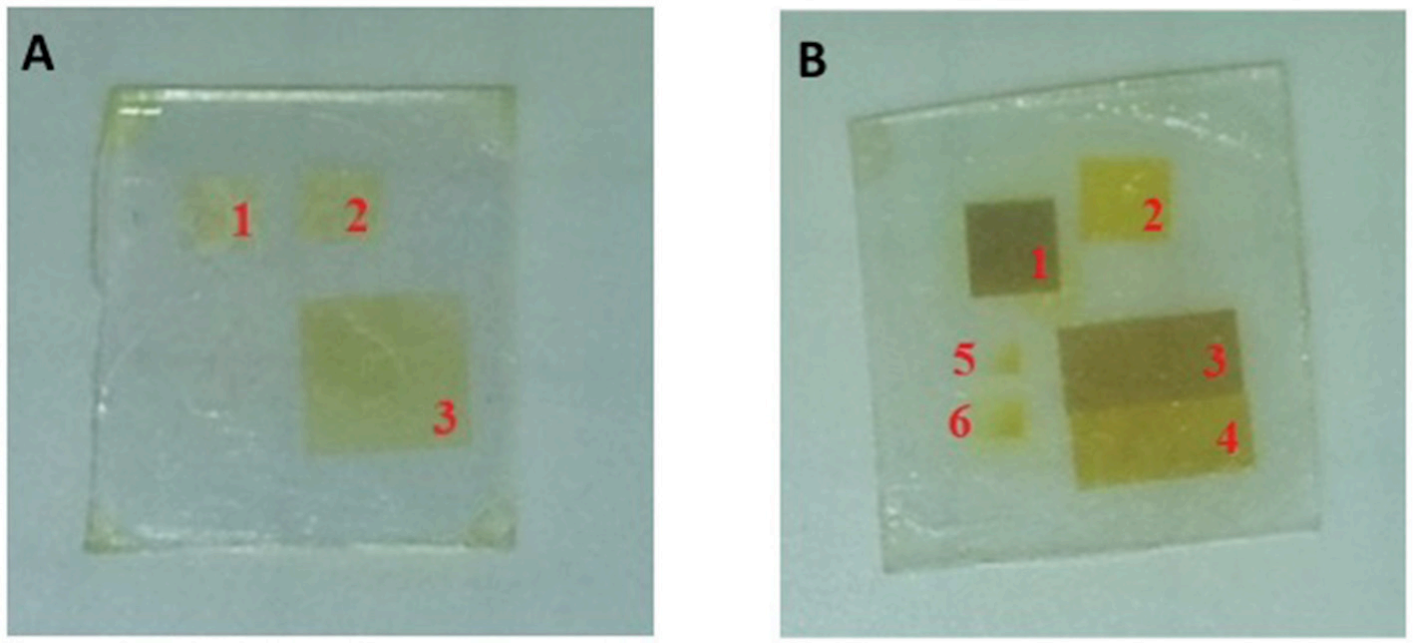
**(A)** Cadmium dibuthylxanthate film irradiated with a 266 nm, 10 ps, 100 kHz laser source at different average power: 1–25, 2–30, 3–35 mW. **(B)** CdMASe film irradiated with a 266 nm, 10 ps, 100 KHz laser source at different average power: 1–20, 2–8, 3–15, 4–10 mW, 5-6 dot matrix tests.

The xanthate molecules were tested as materials for laser patterning and the preliminary results showed the formation of QDs from cadmium dibuthyl xanthate (Račiukaitis et al., 2013). In this case, by changing the energy dose of the laser, it is possible to modulate the color of the precursors from faint yellow to deep yellow suggesting that the QD's size can be modulated by the deposited energy. (Figure 6A). However, the CdS QDs are not the best candidates for the luminescent emission even when produced by solvothermal methods due to the presence of many surface defects (broadening of PL emission spectrum) and low emission in the visible range. The CdSe QDs were therefore explored as new candidates, because they show better optical properties, including less surface defects and a wider spectrum of emission in the visible spectral range (Regulacio and Han, 2010). These characteristics enable their use in light emitting devices like QD-LEDs.

**Figure 7:**
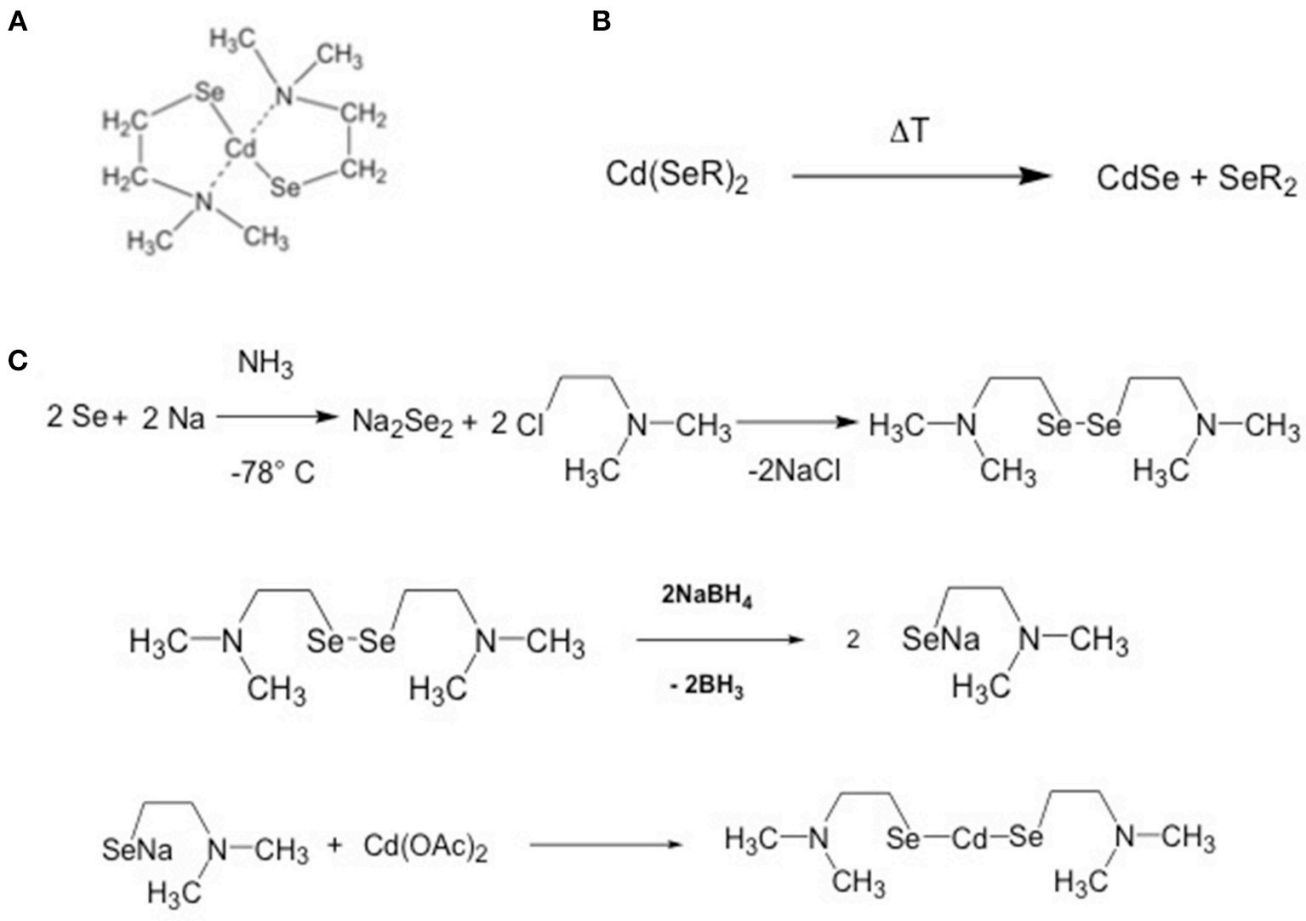
**(A)** chemical structure of the CdDMASe **(B)** formation of the CdSe QDs after backing; **(C)** scheme of the synthesis of the CdDMASe.

The criteria for the selection of the precursors of CdSe QDs were the same as for CdS QDs: (i) solubility in organic solvents, (ii) decomposition temperature below 300°C, and (iii) a relatively easy protocol of synthesis and compound stability. This condition will be an important factor for cost reduction in case of industrial applications. A single source CdSe QDs precursor, which combines all these characteristics, is the cadmium 2-(N,N′ dimethylamino) ethylselenolate (CdDMASe, Figure 7) (Kedarnath et al., 2006).

This precursor has been successfully used for the laser induced synthesis of QDs alone (Račiukaitis et al., 2013) and in combination with a small organic molecule like TPBI (Bansal et al., 2015). Račiukaitis et al. showed a correlation between the laser energy dose and the average size of the QDs. The first qualitative evidence was the formation of different colors under laser treatment at 266 nm when the CdMASe is deposited as a neat film. As the laser power increases the color of the film becomes darker: the more reddish/brown color is correlated with a higher amount/size of the CdSe QDs (Figure 6B). The change of the QDs size as a function of the laser energy was revealed by TEM measurement when CdSe QDs were grown in combination with a polyfluorene like film. Table 2 shows that the CdSe QDs grown by laser form clusters containing 10–20 QDs and their size increases from 6 to 10 nm when the delivered energy is enhanced, as expected.

**Table 2:** QDs parameters in Polyfluorene derived polymer/CdDMASe film after ps-laser treatment at 266 nm (With permission of Račiukaitis et al., 2013).

**Power (mW)**	**Scan speed (mm/s)**	**Cluster size (number of QDs)**	**QDs size (nm)**
20	1.0	10–20	6.0 ± 1.1
35	1.0	10–30	10.2 ± 4.4

**Figure 8:**
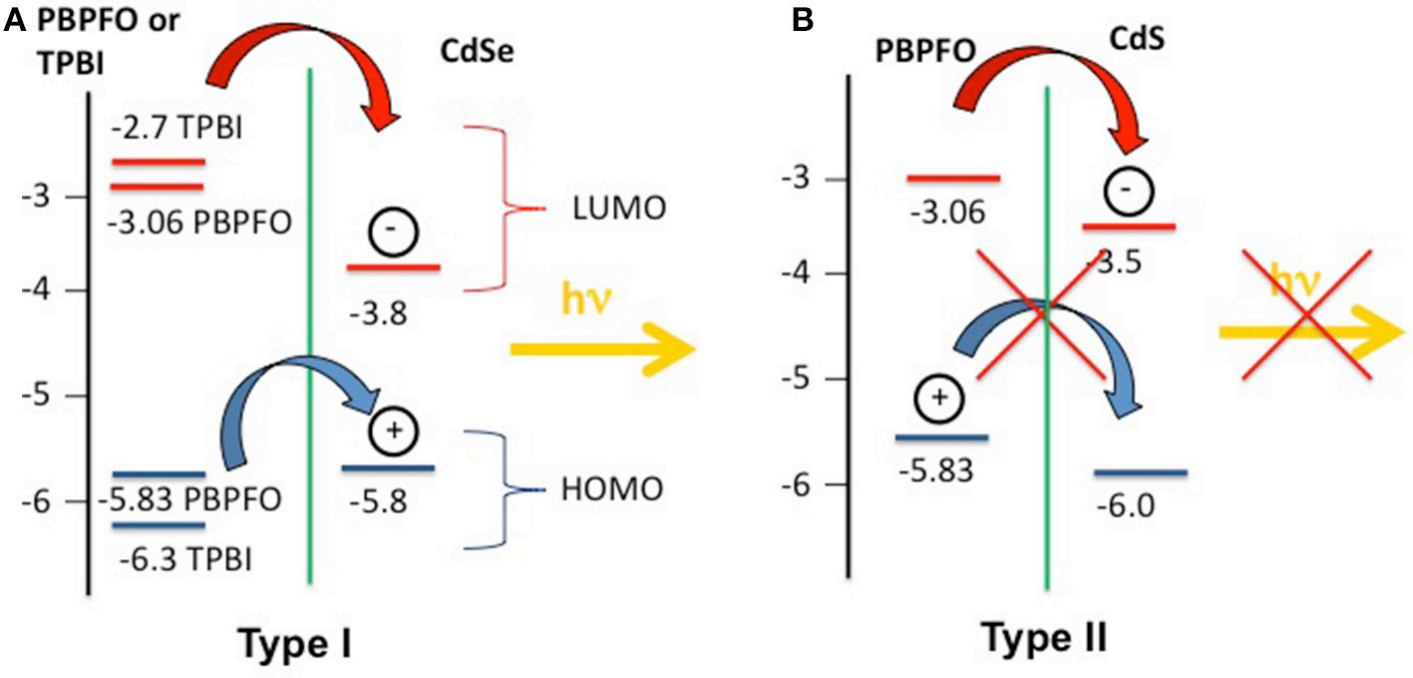
**(A)** The type I system formed by PBPFO and CdSe and TPBI/CdSe energy levels. In the so called Type I configuration the HOMO-LUMO levels of the CdSe QDs are encompassed by the HOMO-LUMO levels of the PBPFO or TPBI. **(B)** The type II system formed by PBPFO and CdS energy levels. When the HOMO-LUMO levels of the CdS and PBPFO are staggered the resulting system belong to the so called Type II system.

The Samuel research group used the same precursor coupled with electroluminescent polymers, such as polyfluorene and TPBI, to study the CdSe QDs growth after thermal (Bansal et al., 2014) and laser (Bansal et al., 2015) treatment. The study of the PL spectra from the polyfluorene/CdSe QDs and of the polyfluorene/CdS QDs showed that the PL emission is strongly dependent from the arrangement of the HOMO-LUMO levels of the polymer and the nanocrystals (Todescato et al., 2016). In particular the CdSe/polyfluorene system (Bansal et al., 2014) is photoluminescent, but its CdS counterpart is not emissive. This behavior is due to the arrangement of the HOMO-LUMO energy levels of the polyfluorene/CdSe, that allows the exciton shift from the polymer to the QDs (Type I system) with the emission of one photon (Figure 8A). On the contrary the polyfluorene/CdS system did not show any QDs emission because the HOMO-LUMO levels are arranged like a Type II system, where the hole and electron are displaced in two different materials so that the charge recombination cannot take place (Figure 8B).

**Figure 9:**
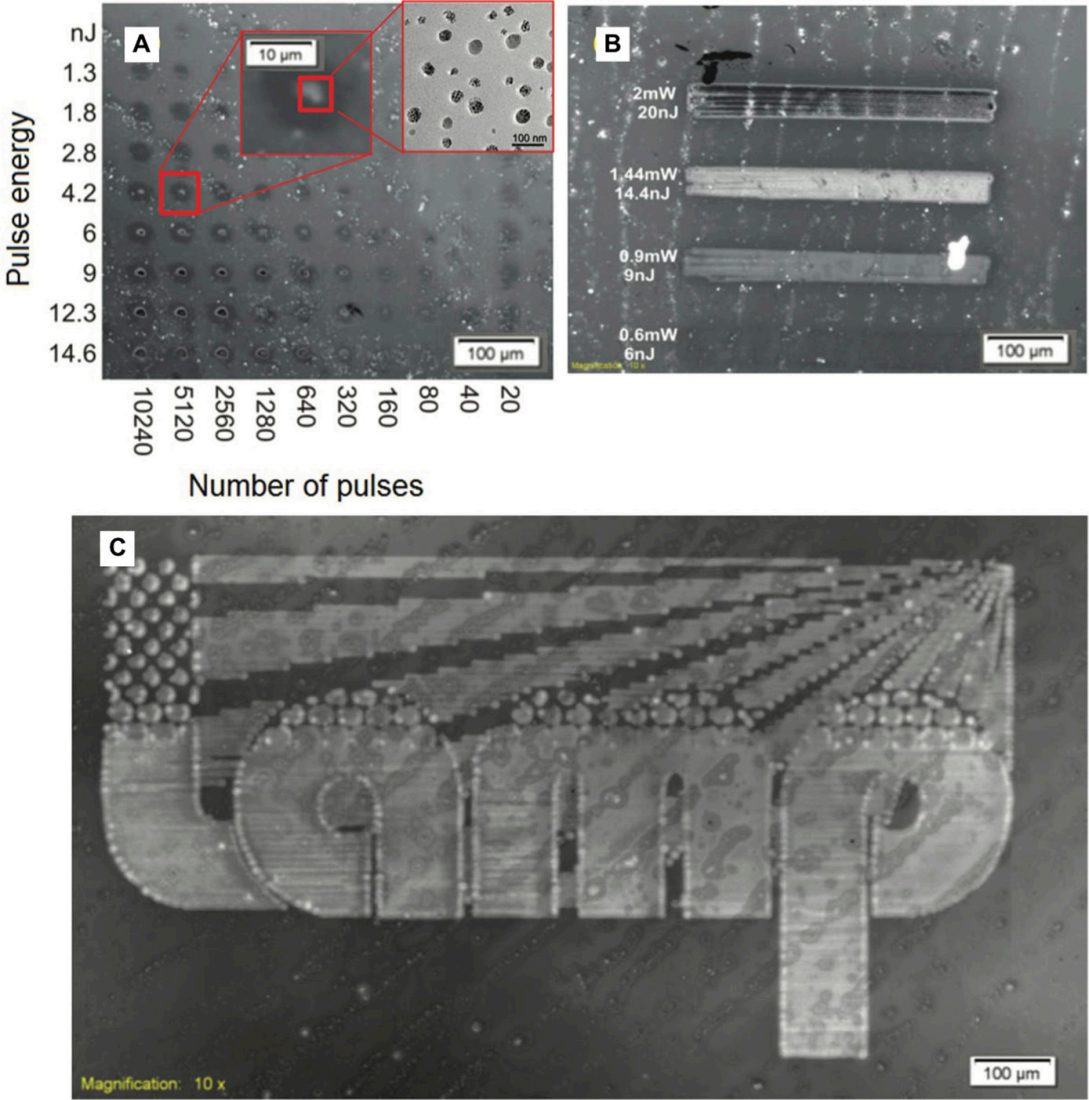
Laser fluorescence image of **(A)** dot matrix to find the optimal conditions of the laser source; **(B)** laser scanning at different mean power, and **(C)** scanning of a logo by using 1.05 mW average power, 100 KHz, 10.5 nJ, and a speed of 0.75 mm/s laser parameter (Bansal et al., 2015).

This evidence is even more pronounced when the TPBI matrix is used instead of polyfluorene, as shown by Bansal et al. (2015). In this case the distribution of the HOMO-LUMO levels of CdSe QDs and the organic matrix is still more favorable for the energy transfer between TPBI and CdSe. In this matrix the photo-luminescent quantum yield (PLQY) of the CdSe QDs increases up to 15.5% (annealing at 160°C for 15 min). With this new system finally it was possible to obtain the visible PL emission from the laser patterned QDs (Figure 9). The best laser patterning conditions were found on the basis of the PL response. This was done by building a matrix of pulse energy vs. number of pulses (Figure 9A), then selecting the best speed of patterning (Figure 9B) and finally drawing the logo (Figure 9C). The emission properties of the patterned areas are similar to the ones obtained with backing.

The mechanism of the QDs formation is still under investigation. However, the use of UV laser (266 nm and 355 nm) radiation, that is absorbed by the CdS and CdSe precursors, points toward a photochemical breaking of the carbon and chalcogenide bonds and then favors the reorganization of the atoms to form the nanocrystal. The Bityurin group (Smirnov et al., 2018) has recently deeply investigated the mechanism of formation of the CdS QDs from a precursor under thermal or photochemical activation. Here a dithiobiuret complex of cadmium embedded in the PMMA matrix is decomposed under a UV light treatment at different temperatures. The UV radiation helps the formation of the QDs and the lack of results under furnace treatments at the same temperature suggests that the QDs growth is mainly a photochemical process rather than a thermal process. This means that the UV lasers can affect the synthesis also by the interaction of the energetic photons directly on the precursor.

Recent results about QDs synthesis by using a 532 nm laser has been presented by Morselli et al. (2016). Here the used precursor is the zinc acetate for the production of zinc oxide after laser treatment. The authors reported the formation of the ZnO QDs with unknown volatile compounds being formed as a by-product, but the chemistry of the process is still not clarified.

Another strategy to pattern QDs using lasers is to modify the adhesion characteristic of the substrate with respect to the QDs (Chen et al., 2017b). A two stage process was demonstrated by selectively patterning perovskite based QDs on glass surface. The process involved a 405 nm UV continuous laser and a following washing step to remove, by dissolution, the untreated QDs and obtaining patterned regions. However, in this approach, the QDs are prepared “*ex situ*” and deposited over a substrate and the role of the laser is to modify only the adhesion of the QDs over the substrate and not their synthesis. Indeed, the laser action removes the organic ligands around the QDs surface modifying their ability to bind the surface. In general, the effect of the laser is 2-fold: first it decreases the PL emission with respect to the untreated perovskite QDs and, secondly, the laser fluence causes also the QDs size modification.

### Laser Patterning of QDs: The Effect of the Pulse Duration and Wavelength

The examples in literature to induce QDs formation/modification by laser can be categorized by time operating regime: from continuous wave to the femtosecond range, and by laser wavelength: from near infrared (NIR) solid state laser to fourth harmonic UV laser. This broad range of patterning methods suggest that different physical phenomena drive the process, from thermal annealing to “cold” photochemical process.

About wavelength, the first example of QDs generation was obtained with a high power Nd:YAG using 10 to 50 ms pulses with an estimated fluence of about 16 kW/cm^2^ (Antolini et al., 2006). Such high fluence had to cause the complete degradation of the polymer film; in order to explain the reported successful results, the very low absorptivity of polystyrene matrix at the 1,064 nm wavelength of Nd:YAG laser should be taken in account. Moreover, the used NIR laser can be considered as a local heating source and the process is essentially a thermal one, like in Hu and Wu (2013).

**Figure 10:**
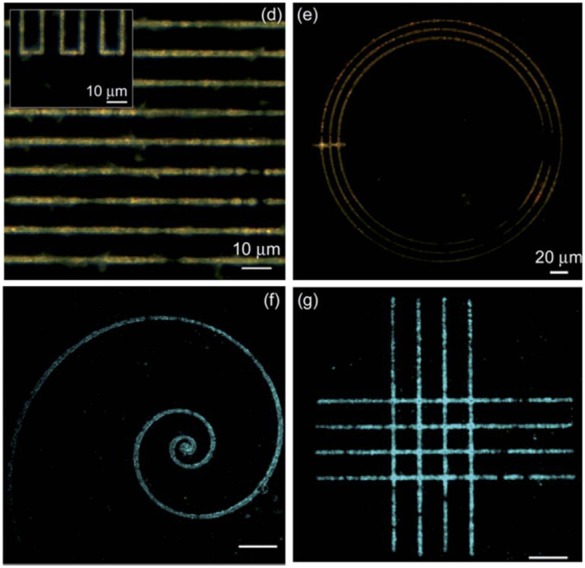
Different geometric patterns obtained by Camposeo et al. (2012) scanning at 0.2 mm/s with an incident fluence of 0.2 J cm^−2^ (With permission of Camposeo et al., 2012).

Another example of the use of near IR lasers is presented in Camposeo et al. (2012), where a 200 femtoseconds laser operating at about 800 nm is used. In this case the absorption of precursors in the near IR is almost negligible and authors suggest that a multiphoton absorption mechanism take part in the growth of QDs. In any case, the use of an ultrashort laser avoids thermal effects and permits, thanks to the use of an oil-immersion large aperture objective, the ability to generate very fine and detailed patterns with micrometric resolutions (Figure 10).

An example of the use of a second harmonic continuous Nd:YAG laser to generate QDs in solution is presented in Lin et al. (2005). In this work, high-quality CdSe QDs were prepared by irradiating an aqueous solution at 532 nm, the same wavelength was used in Morselli et al. (2016) to synthesize ZnO nanoparticles in PMMA/Zn(OAc)_2_ films. Even though these nanoparticles were not generated to operate as QDs, the results demonstrates the possibility to pattern a film with a laser operating at 532 nm, as already shown in Qayyum et al. (2016).

Račiukaitis et al. (2013) and Singh et al. (2018) operated in the UV range. In particular Račiukaitis et al. (2013) investigated the effects of the 266 nm fourth harmonic UV picosecond laser patterning of polymer/precursor blends, obtaining color change in the laser treated areas of precursors and QDs size modulation as function of laser fluence as discussed in the previous paragraph.

In (Singh et al., 2018) QDs are generated in TGA capped CdTe using the second harmonic of a Ti-sapphire laser. The authors described the QDs generation as a thermal process, but in this case the medium is liquid and QDs generation is strictly connected with the use of the laser wavelength in the UV range (380–415 nm), while no effects were measured using the first harmonic (800 nm). This behavior suggests the presence of a photochemical phenomenon rather than a thermal one.

**Figure 11:**
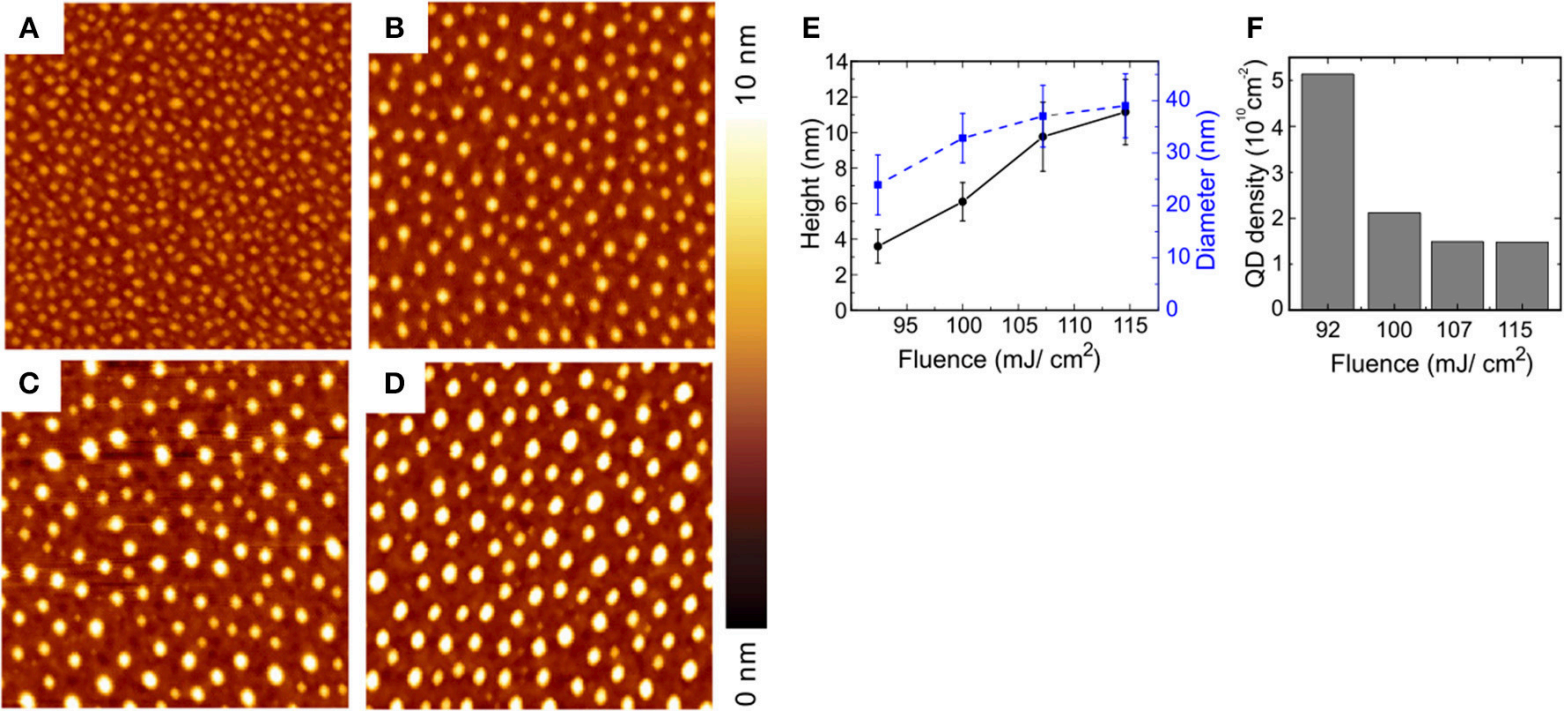
Laser induced Ge/Si QDs obtained by patterning a thin (0.9 nm) Ge coating on Silicon substrate with different fluences, **(A)** 92 mJ/cm^2^, **(B)** 100 mJ/cm^2^, **(C)** 107 mJ/cm^2^, **(D)** 115 mJ/cm^2^. The dimension of AFM maps is 1 × 1 μm (With permission of Qayyum et al., 2016).

Laser induced QDs generation in a different matrix was demonstrated in Voznesenskiy et al. (2015) in which CdS QDs in porous silica was successfully modified changing their dimensions by agglomeration when irradiated at fluence higher than 0.1 J/cm^2^ at 405.9 nm. The process effectiveness depends on the right choice of laser parameters. Moving to other semiconductors, in Qayyum et al. (2016) authors show the generation of uniformly distributed Ge/Si QDs by laser patterning of a Ge coated Si substrate with the second harmonic at 532 nm of a Nd:YAG laser with a pulse duration of 10 ns. In this case the higher absorptivity of Ge compared to Si at this wavelength opens an operating window between 92 and 115 mJ/cm^2^ in which very uniform and densely distributed QDs are generated with mean diameters between 20 and 40 nm and height between 4 and 10 nm, (Figure 11). The authors proposed a simple phenomenological model of the QDs formation under these experimental conditions.

### Pulse Duration

About the time distribution of the laser intensity, modern laser sources permit operation from the continuous regime to Q-switched pulsing (ns regime) to mode locking (ps and fs regimes). All these operating regimes can be used to generate QDs.

#### Continuous Regime

Continuous regimes as in Lin et al. (2005) and long pulses regime as in Antolini et al. (2006) for QDs generation in aqueous solutions and for the generation of QDs starting from precursors embedded in thick polymer films, respectively, are examples of laser induced thermal activation. In these cases, the temperature is locally raised by heating the material, thanks to the absorption of the laser intensity in the film thickness. The absorption, assuming that the laser scattering is negligible, will follow the general formula:

$$I(x,y,z,t)=(1-R)\;I_{0}(x,y,t)(1-e^{-\alpha z})$$

where *I*_0_(*x, y, t*) is the laser intensity spatial and temporal distribution on the material surface expressed in *W*/*m*^2^, *z* is the depth in the polymer thickness in *m*, *R* is the film reflectivity at the laser wavelength and α is the material absorptivity expressed in *m*^−1^.

The heat flux absorbed by the material under patterning is distributed by conduction, following the usual Fourier equation:

$$C_{P}\;\rho \;\frac{\partial T}{\partial t}=\frac{\partial }{\partial x}\left(K\frac{\partial T}{\partial x}\right)+\frac{\partial }{\partial y}\left(K\frac{\partial T}{\partial y}\right)+\frac{\partial }{\partial z}\left(\;K\frac{\partial T}{\partial z}\right)+\alpha I(t)$$

in which *C*_*P*_ is the specific heat in *J kg*^−1^ *K*^−1^, ρ is the material density in *kg*/*m*^3^, *K* is the thermal conductivity in *W m*^−1^ *K*^−1^ and α *I* is the heat source in *W*/*m*^3^.

Selective patterning with long pulses or in continuous wave mode is strongly influenced by thermal diffusivity and under these regimes fine details cannot be obtained.

#### Q Switched Regimes

Short pulses in the ns range can be obtained with Q-switch modulated laser. In Athanassiou et al. (2007) CdS QDs in TOPAS are generated by the use of XeCl excimer laser at 308 nm, pulse duration of 30 ns, fluence of 100 *mJ*/*cm*^2^, and a repetition rate of 1 Hz. A strong correlation between the number of irradiation pulses and PL emission (from 490 to 501 nm) was observe and confirmed that larger QDs size growth when the deposited energy is increased. The correlation observed also between the total number of pulses and the PL intensity indicated that the increase of the pulses number enhances the emission broadening and the surface defects.

Fragouli et al. investigated both the effects of polymer matrix (Fragouli et al., 2010a) and laser wavelength (Fragouli et al., 2010b) on the formation of CdS nanocrystals in PMMA and TOPAS matrix. In particular, an 8 ns pulsed Nd:YAG laser operating at third (355 nm) and fourth harmonic (266 nm) was implemented, cumulating pulses to tune the QDs emission as already discussed (section Materials for direct laser patterning of QDs).

The use of laser induced generation of nanoparticles was investigated also in Onwudiwe et al. (2014a) for ZnS in polyvinylpyrrolidone and in Onwudiwe et al. (2014b) for CdS in PVA.

**Figure 12:**
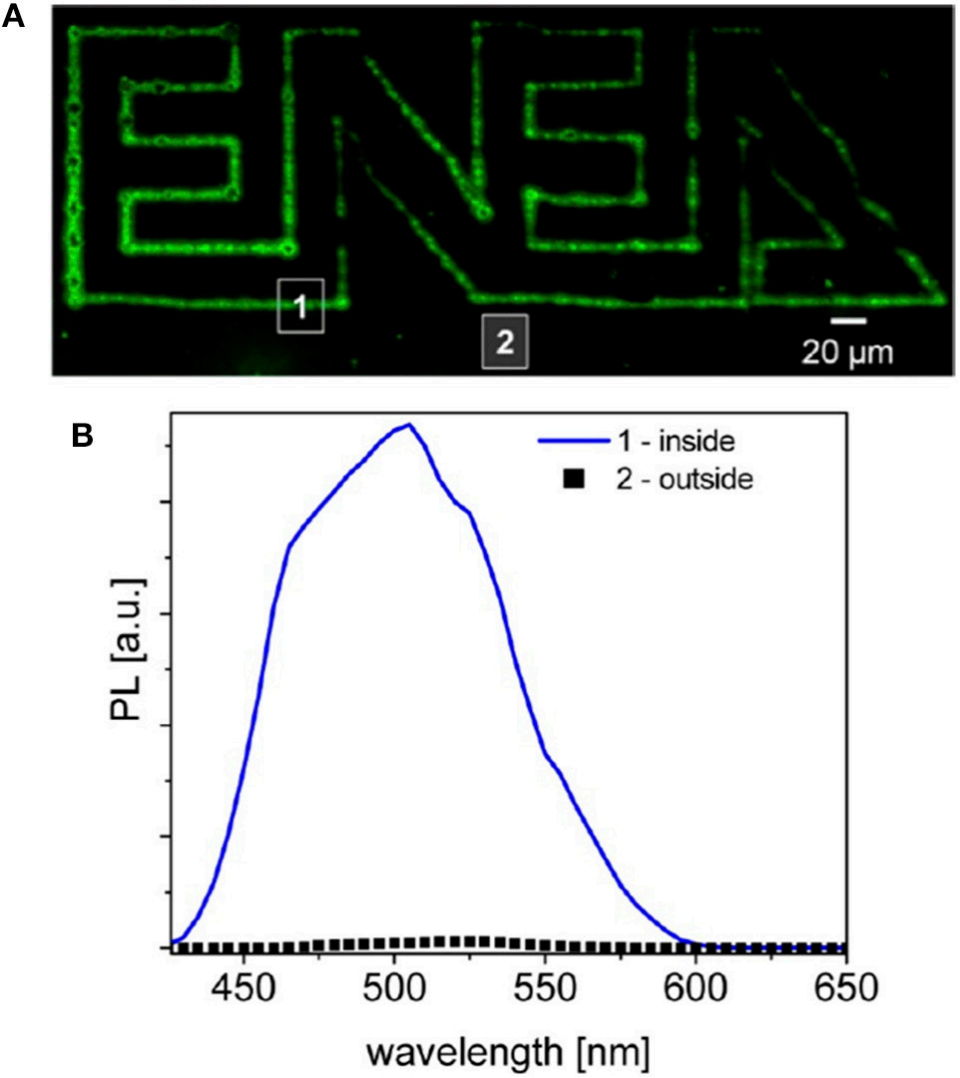
Resta et al. (2012) Laser processing of [Cd(SBz)_2_]_2_·MI/PMMA with a fluence of ~ 700 *mJ*/*cm*^2^. In **(A)** the fluorescence emission of the patterned polymer under excitation at 405 nm. In **(B)** the spectral emission (With permission of Resta et al., 2012).

While in the previous works the accuracy and the resolution attainable by using short pulsed laser were not investigated by operating in liquid phase, in Resta et al. (2012) a PMMA polymeric film with CdS precursor was selectively patterned with a third harmonic laser (λ = 355 nm, τ = 10 ns) and a minimum focused spot of 10 μm (Figure 12).

#### Mode Locking Regimes

In recent years the ultrafast lasers operating in mode-locking has become cheaper, more compact, more reliable and highly operable, thus opening a broad range of scientific, industrial, and medical applications recognized also by the 2018 Nobel prize in physics (The Nobel Prize in Physics, 2018).

**Figure 13:**
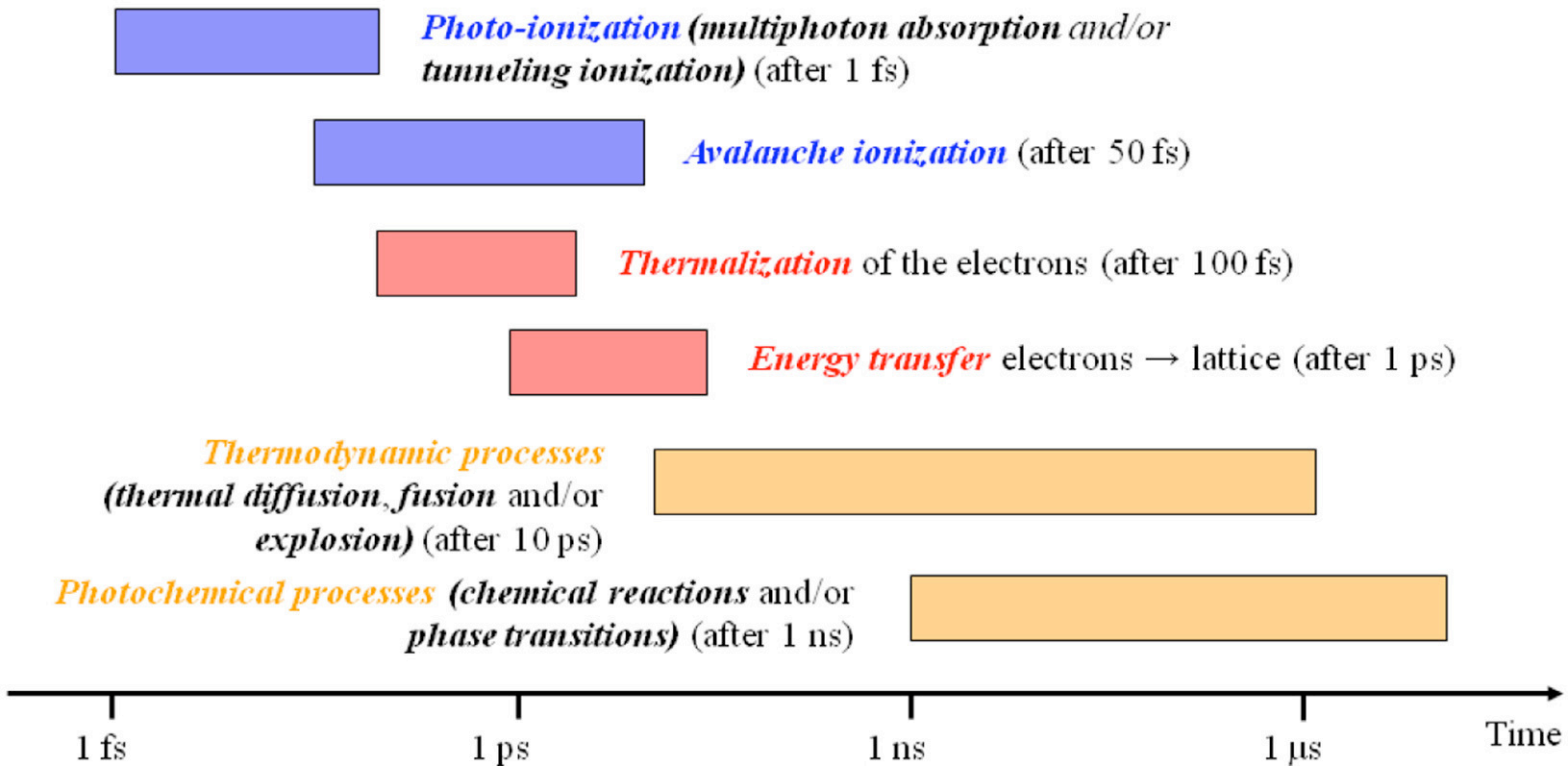
Timescales in laser matter interaction, adapted from Royon et al. (2011) (With permission of the authors).

Laser sources with pulse duration shorter than few picoseconds are currently available for both scientific and industrial applications. Under this operating regime light/matter interaction is completely different from what is seen in continuous or short pulse lasers. A scheme of the laser/matter interaction at the different timescales is presented in Figure 13, from Royon et al. (2011) below 1 ps no energy is transferred from ionized/thermalized electrons to the molecules in the solid lattice, and thermal diffusion only starts to operate after about 10 ps. Until this point, the temperature of electrons and the lattice are different and hence the system must be described by the so-called two temperature model (Anisimov et al., 1974).

$$\begin{array}{l} C_{e}\frac{\partial T_{e}}{t}=\frac{\partial }{\partial x}\left(K_{e}\frac{\partial T_{e}}{\partial x}\right)+\frac{\partial }{\partial y}\left(K_{e}\frac{\partial T_{e}}{\partial y}\right)+\frac{\partial }{\partial z}\left(K_{e}\frac{\partial T_{e}}{\partial z}\right)\\ \;+\Gamma \left(T_{l}-T_{e}\right)+\alpha I\left(t\right)\\ C_{l}\frac{\partial T_{l}}{\partial t}=\frac{\partial }{\partial x}\left(K_{l}\frac{\partial T_{l}}{\partial x}\right)+\frac{\partial }{\partial y}\left(K_{l}\frac{\partial T_{l}}{\partial y}\right)+\frac{\partial }{\partial z}\left(K_{l}\frac{\partial T_{l}}{\partial z}\right)\\ \;+\Gamma \left(T_{l}-T_{e}\right) \end{array}$$

In this model the laser source α *I* interacts with electrons, the temperature *T*_*e*_ of the electrons and *T*_*l*_ of the lattice will depend by the respective conductivities *K*_*e*_ and *K*_*l*_ and by Γ(*T*_*l*_−*T*_*e*_), the electron-lattice coupling term that describe the heat flux between electrons and lattice. In this classical model the conduction in lattice can be normally neglected compared to electrons *K*_*e*_≫*K*_*l*_. The pulse duration of 10 ps can be considered a general threshold for the ultrashort pulsed regime, in which both physical and chemical effects on molecules take part after the end of the irradiation, and the normal pulsed regime, where molecules and lattice transformations occur during the pulse duration. By operating below this pulse duration, it is possible to obtain a high spatial accuracy, theoretically below the diffraction limit if the process threshold is close to the maximum intensity in the center of the laser spot.

As already cited (Camposeo et al., 2012) proposed the use of a very short 180 fs laser at 800 nm to achieve direct laser writing of high resolution patterns of CdS QDs in TOPAS®-C12 films. Three-dimensional silver nanostructures with dimensions of nanometers have also been patterned by Vora et al. (2012) in polyvinylpyrrolidone with the use of a 50 fs Ti:sapphire laser operating in the first harmonic.

Račiukaitis et al. (2013) operated with the IV harmonic at 266 nm, 10 ps pulse, of a .Nd:YAG laser generating QDs in electroluminescent polyfluorene like polymers films, and they were able to determine the relationship between QDs size and laser parameters using TEM (see section Materials for direct laser patterning of QDs).

## Concluding Remarks

The direct laser patterning of QDs has a great technological potential because it combines three main concepts:

- Laser light can stimulate growth of QDs in the solid state in selected areas of the irradiated materials;
- The laser light can be suitably modulated (wavelength, pulse energy, repetition rate etc) to tune the particle size;
- The QDs modify their chemical-physical properties as a function of their size/shape.

These three concepts: the “possibility to drive the position of photons,” the “possibility to modulate the energy distribution of the photons,” typical of laser writing process and the “possibility to modulate the photophysical properties of the QDs as a function of their size,” typical of the nanomaterials can be exploited in several fields of the science and technology and in this review is envisaged its possible application in the manufacturing of QD-based displays.

Indeed, in display technologies it is necessary to pattern in selected areas of a surface the red, green, and blue subpixels in order to obtain a RGB matrix that can be realized by using the suitable precursors and laser parameters. In this case the QDs would be conveniently used as color converter materials, as already reported for displays application (Steckel et al., 2015; Chen et al., 2017a).

**Figure 14:**
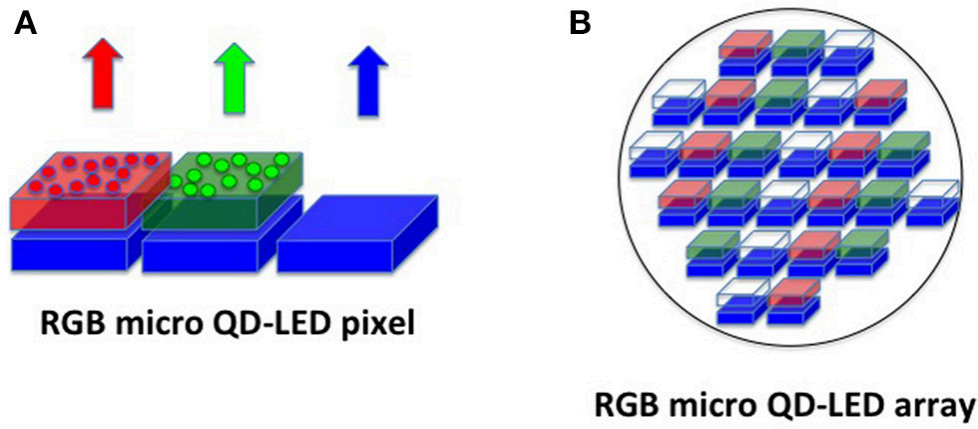
Illustration of the principle for the formation of a RGB pixel driven by three blue micro-LED/OLEDs covered with red and green QDs **(A)**. The manufacturing of a matrix of a large number of blue micro-LED/OLEDs patterned with red and green QDs form a display **(B)**.

A further step ahead can be done considering that recently micro-LED sources become available so that they can be used as RGB pixels in a display (Wu et al., 2018). However, this technology suffers from several disadvantages for mass production, because the main difficulty is to produce green and red micro-LED efficiently and at low prices in the same substrate. A possible solution to cover this so-called “green gap” is to employ a blue micro-LED array and to pattern over it the red and green color converters, constituted by QDs (Han et al., 2015; Lin et al., 2017) generated directly by the laser writing process with high precision and resolution (Figure 14).

These basic concepts, such as blue micro-displays and laser patterning of QDs, are nowadays implemented by the EU project MILEDI, (www.miledi-h2020.eu) for a display manufacturing by using lasers.

It is worth-noting that in this review the direct laser patterning and its technological exploitation was discussed in terms of Cd based QDs because they are the best-developed and well-characterized materials, and their optoelectronic properties cover the entire visible spectrum. Indeed the Cd based QDs benefit from a well-known synthesis route, that allows a PLQY almost at unity with a quite narrow FWHM (20–30 nm) (Christodoulou et al., 2014).

However, the main drawback of these materials is their content of heavy metals, that has been limited by recent regulation, as, for example the EU Directive 2002/95/EC 2003. This limitation was also considered in the recent project MILEDI taking into account, as an alternative to the heavy metal based QDs, the ternary alloys like CuIn(S, Se)_2_ and AgIn(S, Se)_2_, that provide light emission extending in all the visible spectrum up to the NIR.

Finally Scalbi et al. (2017) studied the environmental impact, by means of the Lyfe Cycle Assessment methodology, of a QD-LED including the CdS QDs made by laser and embedded in a electroluminescent polymer like polyfluorene. Surprisingly the final results showed that the hotspots for the environmental impact are the polymer synthesis and ITO electrode manufacturing. Indeed, the use of catalysers such as Palladium and of solvents like the hexane for the synthesis of polyfluorene have a major environmental impact on device life.

These results are encouraging for a further exploration of this approach for display manufacturing by using direct laser patterning methodology and QDs even containing heavy metals.

## Author Contributions

FA is the coordinator of the work and involved LO for the laser patterning section.

### Conflict of Interest Statement

The authors declare that the research was conducted in the absence of any commercial or financial relationships that could be construed as a potential conflict of interest.
